# Vitamin D and Exercise Are Major Determinants of Natural Killer Cell Activity, Which Is Age- and Gender-Specific

**DOI:** 10.3389/fimmu.2021.594356

**Published:** 2021-06-23

**Authors:** Sooyeon Oh, Sukyung Chun, Sena Hwang, Jongseok Kim, Yuri Cho, Jooho Lee, KyuBum Kwack, Sang-Woon Choi

**Affiliations:** ^1^ Chaum Life Center, CHA University School of Medicine, Seoul, South Korea; ^2^ Department of Biomedical Science, College of Life Science, CHA University, Seongnam, South Korea; ^3^ Center for Liver and Pancreatobiliary Cancer, National Cancer Center, Goyang, South Korea; ^4^ Department of Gastroenterology and Hepatology, CHA Bundang Medical Center, CHA University School of Medicine, Seongnam, South Korea; ^5^ School of Public Health and Health Sciences, University of Massachusetts, Amherst, MA, United States

**Keywords:** vitamin D, NK cell activity, exercise, immunosenescence, immunity

## Abstract

**Background:**

The coronavirus-19 disease (COVID-19) pandemic reminds us of the importance of immune function, even in immunologically normal individuals. Multiple lifestyle factors are known to influence the immune function.

**Objective:**

The aim was to investigate the association between NK cell activity (NKA) and multiple factors including vitamin D, physical exercise, age, and gender.

**Methods:**

This was a cross-sectional association study using health check-up and NKA data of 2,095 subjects collected from 2016 to 2018 in a health check-up center in the Republic of Korea. NKA was measured using the interferon-γ (IFN-γ) stimulation method. The association of NKA with 25-(OH)-vitamin D (25(OH)D) and other factors was investigated by multiple logistic regression analysis.

**Results:**

The average age of subjects was 48.8 ± 11.6 years (52.9% of subjects were female). Among 2,095 subjects, 1,427 had normal NKA (NKA ≥ 500 pg IFN-γ/mL), while 506 had low NKA (100 ≤ NKA < 500 pg/mL), and 162 subjects had very low NKA (NKA < 100 pg/mL). Compared to men with low 25(OH)D serum level (< 20 ng/mL), vitamin D replete men (30–39.9 ng/mL) had significantly lower risk of very low NKA (OR: 0.358; 95% CI: 0.138, 0.929; *P* = 0.035). In women, both low exercise (OR: 0.529; 95% CI: 0.299, 0.939; *P* = 0.030) and medium to high exercise (OR: 0.522; 95% CI: 0.277, 0.981; *P* = 0.043) decreased the risk compared to lack of physical exercise. Interestingly, in men and women older than 60 years, physical exercise significantly decreased the risk. Older-age was associated with increased risk of very low NKA in men, but not in women.

**Conclusion:**

Physical exercise and vitamin D were associated with NKA in a gender- and age-dependent manner. Age was a major risk factor of very low NKA in men but not in women.

## Introduction

The coronavirus-19 disease (COVID-19) pandemic caused by SARS-CoV-2 prompted us to keep our immune function healthy to protect the body from the pathogen. Physical exercise, nutrition and emotional status were frequently named as the main factors that can influence the immune function. Thus, lifestyle that includes regular physical exercise, healthy dietary and emotional habits were emphasized besides wearing masks and social distancing (1–5).

Immune markers that can represent immune function in immuno-competent individuals is still under search. In this regard, earlier research tried to elucidate immune characters that can predict healthy longevity. Decreased immune responses represented by CD4+:CD8+ ratio (<1), poor T-cell proliferation response to mitogens *in vitro*, increased number of CD8+ T lymphocytes and so on were associated with increased mortality in the elderly (6–9). However, those findings could not be reproduced (10, 11). Most of the immune function tests used in clinical practice examine critical functional defects that are present in immunodeficiency diseases. Yet, disease development in immuno-competent individuals is also closely associated with impaired immune function. Overwork, fatigue, and emotional stress are well acknowledged to negatively affect the immune function. However, there are not many tests that can evaluate the immune function in immunologically normal individuals, that is varied by stressors in everyday life. Commercialized natural killer (NK) cell activity (NKA) assay is an immune function test that may fill the need. The NKA test measures the amount of interferon-gamma (IFN-γ) released by activated NK cells contained in 1ml of peripheral blood (12). Therefore, it represents the secretory function of NK cells. Also, it may indirectly indicate the amount of NK cells contained in 1 mL of the peripheral blood approximately. Studies on NKA have demonstrated that decreased NKA is significantly associated with increased cancer incidence in the stomach (13), colorectum (14), and prostate (15, 16).

NK cells, as members of the innate immune system, are early responders of immune reactions. Functional defects of NK cells results in immunodeficiency syndrome which pose the individuals at the risk of critical infections or cancer development (17). Decreased NK cell function in immunologically normal individuals were associated with increased risk for cancer development (13–16, 18, 19). Decreased NK cell function in the immunologically normal elderly was associated with increased risk for severe infection and mortality (20, 21). Further, studies on COVID-19 suggested that NK cell may play a critical role in the early response which can determine overall outcome of the disease. For example, severe COVID-19 cases were characterized by depleted peripheral NK cell counts compared to mild cases or healthy controls (22–26). CD56^dim^ NK cells, which mediate cytotoxicity, were depleted in ventilator-dependent patients, and CD56^bright^ NK cells, that is immune-regulatory producing IFN-γ, were significantly depleted in all COVID-19 patients (23).

The traits of NK cells and the NKA test suggest that the NKA may serve as a useful surrogate marker to evaluate immune function of the immunologically normal individuals. We postulated that there exist lifestyle characteristics which define better immune function. Immune function is affected by multiple factors such as age, gender, nutrition, exercise, and underlying diseases. We investigated how each factor was associated with the NKA.

## Methods

### Study Population

**Figure 1:**
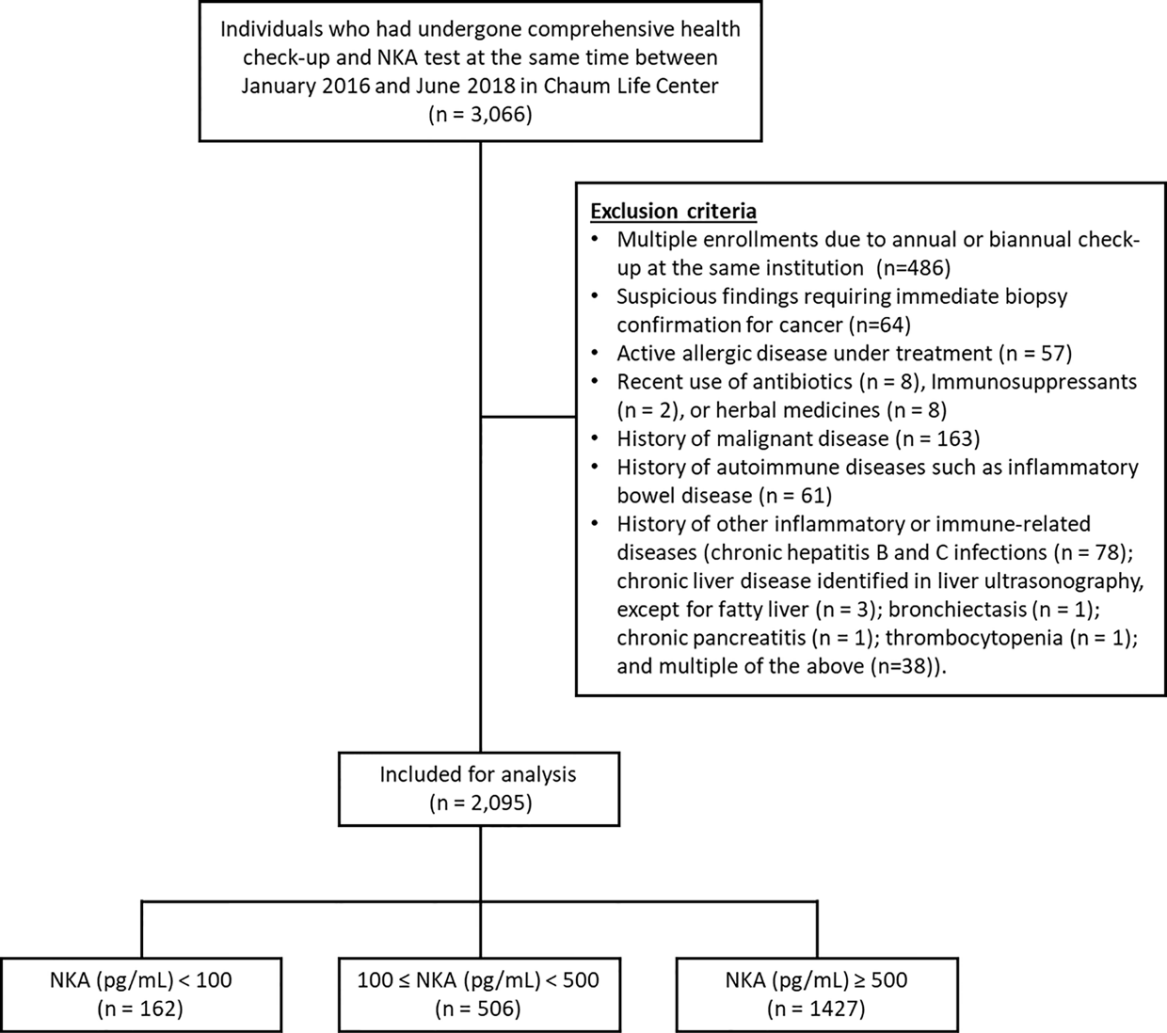
Participant flow chart. NKA, natural killer cell activity.

Between January 2016 and June 2018, individuals aged ≥ 18 years (n = 3,066) who had undergone a comprehensive health check-up and NKA test at Chaum Life Center (Seoul, Republic of Korea) were screened (**Figure 1**). For the enrolled individuals, health check-up data were retrieved from electronic medical records. A certain proportion of individuals had the health check-up annually or biannually at the same institution, which resulted in multiple enrollments. In such case, only the first check-up data were included, and the check-up data of the following years were excluded (n=486). Exclusion criteria included suspicious findings in health check-up results requiring immediate biopsy confirmation for cancer (n = 64), active allergic disease under treatment (n = 57), recent use of antibiotics (n = 8), immunosuppressants (n = 2), or herbal medicines (n = 8), and history of malignant disease (n = 163), autoimmune diseases such as inflammatory bowel disease (n = 61), and other inflammatory or immune-related diseases [chronic hepatitis B and C infections (n = 78); chronic liver disease identified in liver ultrasonography, except for fatty liver (n = 3); bronchiectasis (n = 1); chronic pancreatitis (n = 1); thrombocytopenia (n = 1); and multiple of the above (n=38)]. Individuals with missing variables were automatically excluded during statistical analyses. This study was approved by the Institutional Review Board of CHA Bundang Medical Center (IRB number 2018-06-033-004) and was conducted according to the Declaration of Helsinki. As a retrospective study, the requirement of informed consent was waived.

### Lifestyle Factors

Every individual undergoing health check-up was asked to fill out a questionnaire sent by mail 2 weeks before and to submit it on arrival. The questionnaire asked medical and social history and lifestyle habits including alcohol intake habits, smoking habits and physical exercise habits. Regarding physical exercise habits, it asked about exercise frequency (no exercise, 2–4 times per month, 2-4 times per week, or everyday) and exercise duration per workout session (less than 1-h, 1–2-h, or more than 2-h).

For alcohol intake habits, individuals who did not drink at all or those who drank once a month or less frequently with a limit of 1–2 standard servings per session were defined as non-drinkers. Individuals who drank up to 3 times per week with a limit of 1–2 standard servings per session were defined as light drinkers. Individuals who drank more than light drinkers either in frequency or quantity were defined as heavy drinkers (27–29).

For exercise habits, medium exercise was defined as exercise 2–4 times per week with 1–2-h duration per workout session or everyday exercise with less than 1-h duration per session (30–32). Low and high exercises were defined as less or more exercise than medium exercise in terms of workout frequency or time per session, respectively.

During the check-up, height and body weight were measured. Body mass index (BMI) was calculated by dividing body weight in kilograms by height in square meters. BMI was categorized with cut-offs of < 18.5 kg/m^2^ (underweight), 18.5–27.5 kg/m^2^ (normal to overweight), and ≥ 27.5 kg/m^2^ (obese), as suggested for Asian populations by the World Health Organization (33).

### Laboratory Measurement

NKA was measured using the NK Vue^®^ kit (ATGen, Seongnam, Republic of Korea) containing a recombinant cytokine that specifically activates NK cells to release interferon-γ (IFN-γ) (12). According to the manufacturer’s instructions, 1 mL of peripheral blood was directly added to the test kit and incubated for 20–24 h at 37°C. Upon completion of incubation, the supernatant was collected and centrifuged at 3,000 × *g* for 3 min. The final supernatant was measured for IFN-γ content in pg/mL using enzyme-linked immunosorbent assay. This study used reference ranges provided by the test-kit manufacturer (13–16) and defined NKA < 100 pg IFN-γ/mL as very low, 100 pg/mL ≤ NKA < 500 pg/mL as low, and NKA ≥ 500 pg/mL as normal. Electrochemiluminescence-binding assay based on competition principles (Roche Diagnostics GmbH, Mannheim, Germany) was used to measure serum level of 25-(OH)-vitamin D (25(OH)D). Health check-up factors, including NKA, 25(OH)D, and Hb A1c, were measured at the Department of Laboratory Medicine, CHA Gangnam Medical Center (Seoul, Republic of Korea).

### Statistical Analysis

For univariable analyses, continuous variables were assessed using Student’s *t*-test or analysis of variance (ANOVA), and categorical variables were examined by Pearson’s Chi squared test. To identify the risk factors for or protective factors against low NKA, we compared the very low NKA group with the normal NKA group using multiple logistic regression analyses. Variables were eligible for entry into a multiple logistic regression model if they were significantly associated with *P* < 0.1 in univariable analyses or clinically important. Multiple logistic regression analyses were performed in gender, age, and exercise groups to find differential influences of relevant factors in each subgroup. Correlation was tested with Pearson’s correlation test. IBM SPSS statistics version 21 (IBM Corp., Armonk, NY, USA) was used for all statistical analyses. Box plots were created using the R software (version 3.5.1).

## Results

### Population Characteristics

Among the initially screened 3,066 electronic medical records, 486 records were excluded due to multiple check-ups. Thereafter, 485 subjects were excluded according to the predefined exclusion criteria as already described above. Finally, 2,095 subjects were included for analyses (**Figure 1**).

**Figure 2:**
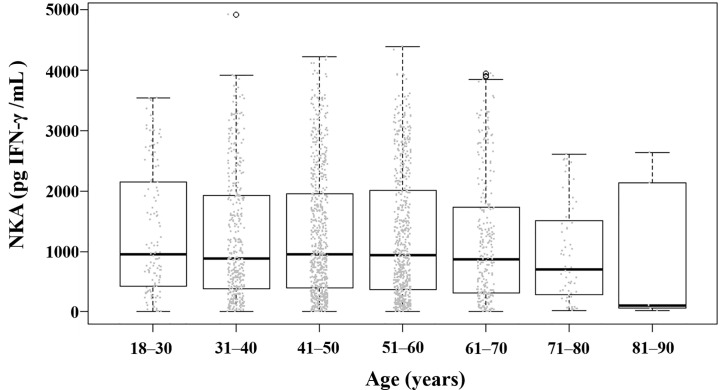
Distribution of NKA value according to age. Each dot represents the individual value of NKA. Box plots show group medians, interquartile range (IQR), and spread of data with outliers for each group. NKA, natural killer cell activity.

**Figure 3:**
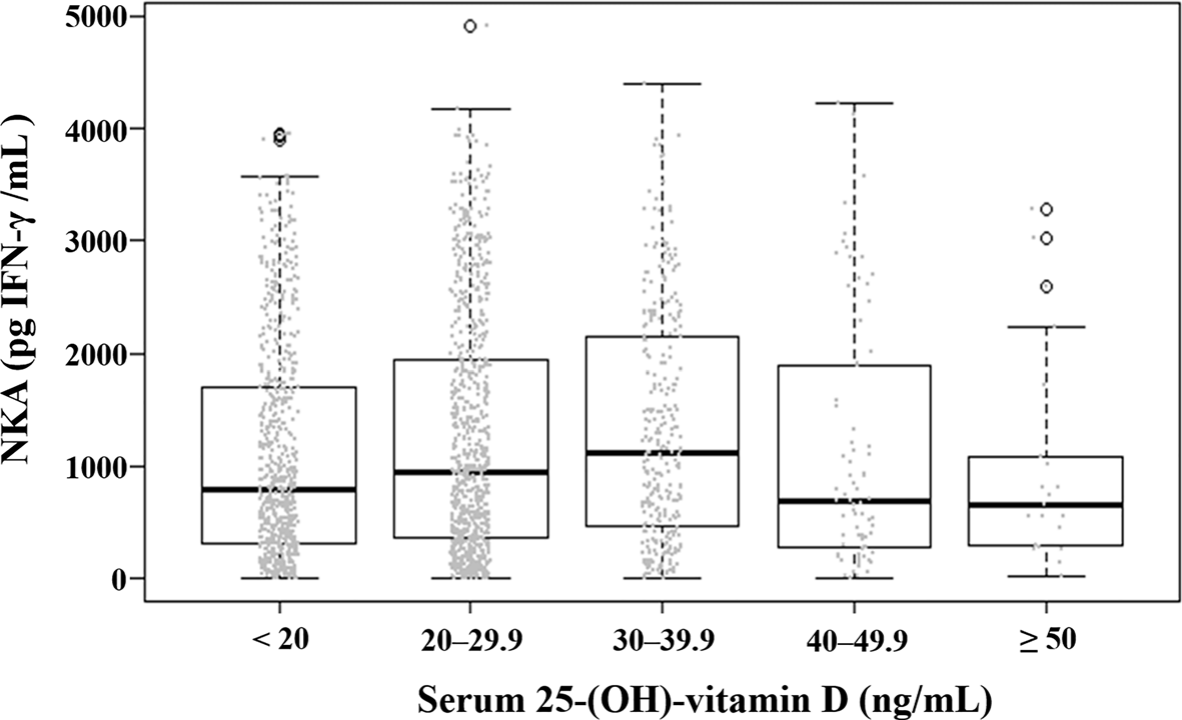
Distribution of natural killer cell activity value according to serum level of 25-(OH)-vitamin D. Each dot represents the individual value of natural killer cell activity. Box plots show group medians, interquartile range (IQR), and spread of data with outliers for each group. NKA, natural killer cell activity.

The mean (± SD) age of the study population was 48.8 ± 11.6 years, 52.9% were female, and the mean (± SD) NKA value was 1,243 ± 1,044 pg INF-γ/mL. Population characteristics according to NKA values are provided in **Table 1**. There were 162 subjects with very low NKA value (NKA < 100 pg/mL), 506 with low NKA (100 ≤ NKA < 500 pg/mL), and 1,427 with normal NKA (NKA ≥ 500 pg/mL). The mean ages of groups with very low NKA, low NKA and normal NKA were 50.72 ± 13.01, 49.19 ± 11.76 and 48.47 ± 11.35 years, respectively (*P* = 0.047). In the very low NKA and low NKA group, the proportions of uncontrolled blood glucose level represented by Hb A1c ≥ 7.5 were higher than the normal NKA group (4.4%, 3.5% *vs.* 1.5%, *P* = 0.034). In the very low NKA and low NKA group, the proportions of vitamin D deficiency represented by serum level of 25(OH)D < 20 were higher than the normal NKA group though it could not reach statistical significance (52%, 48.9% *vs.* 44.4%, *P* = 0.092). The distributions of NKA value according to age and 25(OH)D are depicted in **Figures 2**, **3** respectively. Gender, smoking habit, alcohol use, BMI, hypertension, hyperlipidemia and diabetes mellitus (DM) were not associated with NKA.

**Table 1:** Population characteristics according to NKA.

Characteristics		Very low NKA (NKA < 100 pg/mL, n = 162)		Low NKA (100 pg/mL ≤ NKA< 500 pg/mL, n = 506)		Normal NKA(NKA ≥ 500 pg/mL, n = 1427)		*P* value^1^	*P* value^2^
Age (years, mean)		50.72	± 13.01	49.19	± 11.76	48.47	± 11.35	**0.047**	**0.036**
Age (years)									
	18-30	10	(6.2%)	25	(5%)	86	(6.0%)	0.119	**0.018**
	31-40	25	(15.4%)	97	(19.2%)	267	(18.9%)		
	41-50	45	(27.8%)	146	(28.9%)	453	(31.7%)		
	51-60	46	(28.4%)	154	(30.5%)	414	(293%)		
	61-70	25	(15.4%)	66	(13.1%)	168	(11.8%)		
	71-80	9	(5.6%)	16	(3.2%)	35	(2.5%)		
	81-90	2	(1.2%)	1	(0.2%)	2	(0.1%)		
Gender									
	Women	86	(53.1%)	272	(53.8%)	750	(52.6%)	0.897	0.898
	Men	76	(46.9%)	234	(46.2%)	677	(47.4%)		
Smoking									
	Non-smoker	85	(54.8%)	272	(55.5%)	743	(53.3%)	0.904	0.839
	Ex-smoker	41	(26.5%)	120	(24.5%)	362	(26.0%)		
	Current smoker	29	(18.7%)	98	(20.0%)	289	(20.7%)		
Alcohol									
	Non-drinker	65	(41.9%)	201	(40.8%)	499	(35.8%)	0.236	0.299
	Light-drinker	31	(20.0%)	92	(18.7%)	290	(20.8%)		
	Heavy-drinker	59	(38.1%)	200	(40.6%)	605	(43.4%)		
Physical exercise									
	Lack of exercise	61	(39.9%)	163	(33.7%)	391	(28.5%)	0.058	**0.026**
	Low exercise	53	(34.6%)	170	(35.2%)	533	(38.9%)		
	Medium exercise	30	(19.6%)	107	(22.2%)	317	(23.1%)		
	High exercise	9	(5.9%)	43	(8.9%)	129	(9.4%)		
Body mass index (kg/m^2^)									
	<18.5	16	(10.0%)	31	(6.2%)	80	(5.7%)	0.301	0.094
	≥18.5, <27.4	126	(78.8%)	409	(81.2%)	1161	(82.2%)		
	≥27.5	18	(11.3%)	64	(12.7%)	172	(12.2%)		
Hb A1c (%)									
	<5.7	102	(63.8%)	321	(63.8%)	901	(63.5%)	**0.034**	0.063
	≥5.7, <6.5	45	(28.1%)	141	(28.0%)	445	(31.4%)		
	≥6.5, <7.5	6	(3.8%)	23	(4.6%)	51	(3.6%)		
	≥7.5	7	(4.4%)	18	(3.6%)	21	(1.5%)		
25-(OH)-Vitamin D (ng/ml)									
	<20	77	(52.0%)	223	(48.9%)	553	(44.4%)	0.092	0.196
	≥20, <30	47	(31.8%)	145	(31.8%)	413	(33.1%)		
	≥30, <40	17	(11.5%)	59	(12.9%)	219	(17.6%)		
	≥40	7	(4.7%)	29	(6.4%)	61	(4.9%)		
Hypertension		23	(14.5%)	76	(15.1%)	192	(13.6%)	0.710	0.768
Hyperlipidemia		20	(12.6%)	56	(11.1%)	184	(13.0%)	0.529	0.867
Diabetes		10	(6.3%)	33	(6.5%)	64	(4.5%)	0.173	0.324

^1^P values were calculated using AVONA or Pearson’s Chi squared test with 3 group variables, NKA (pg/mL) < 100, 100 ≤ NKA < 500 and NKA ≥ 500.

^2^P values were calculated using Student’s t-test or Pearson’s Chi squared test with 2 group variables, NKA (pg/mL) < 100 and NKA ≥ 500.

NKA, natural killer cell activity.

P values in bold denote statistical significance at the P < 0.05 level.

### Vitamin D, Exercise, and NKA

**Figure 4:**
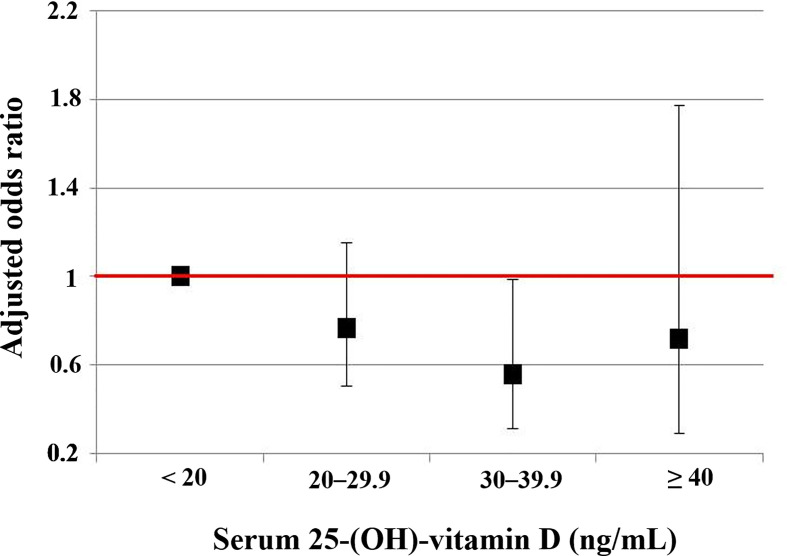
The risks of the very low NKA < 100 pg/mL according to serum level of 25-(OH)-vitamin D compared to normal NKA ≥ 500 pg/mL. This is a result from a multiple regression analysis, and numeric data are shown in the first column of **Table 2**. Vertical lines show 95% confidence intervals.

Compared to 25(OH)D < 20 ng/mL, the 25(OH)D 30–39.9 ng/mL significantly decreased the risk of very low NKA in men (OR: 0.358; 95% CI: 0.138, 0.929; *P* = 0.035). But this association was not observed in women (**Table 2**). The risks of very low NKA in the total population are depicted according to 25(OH)D level in **Figure 4**.

**Table 2:** Factors associated with very low NKA < 100 pg/mL compared to normal NKA ≥ 500 pg/mL.

	Total population		Women		Men	
	OR (95% CI)	*P* value	OR (95% CI)	*P* value	OR (95% CI)	*P* value
Gender						
Women	1 (ref)		–		–	
Men	0.928 (0.649, 1.33)	0.680	–		–	
Age (years)						
18-40	1 (ref)		1 (ref)		1 (ref)	
41-50	1.06 (0.649, 1.74)	0.812	0.882 (0.485, 1.60)	0.680	1.80 (0.701, 4.61)	0.222
51-60	1.25 (0.756, 2.06)	0.387	0.577 (0.289, 1.15)	0.119	3.74 (1.53, 9.12)	**0.004**
61-70	1.75 (0.945, 3.24)	0.075	0.774 (0.322, 1.86)	0.567	5.59 (2.00, 15.6)	**0.001**
≥71	3.26 (1.38, 7.70)	**0.007**	0.940 (0.196, 4.51)	0.938	12.4 (3.62, 42.7)	**<0.001**
Physical exercise						
Lack of exercise	1 (ref)		1 (ref)		1 (ref)	
Low exercise	0.691 (0.454, 1.05)	0.086	0.529 (0.299, 0.939)	**0.030**	0.914 (0.470, 1.78)	0.790
Medium to high exercise	0.551 (0.345, 0.879)	**0.012**	0.522 (0.277, 0.981)	**0.043**	0.612 (0.295, 1.27)	0.188
HbA1c (%)						
<6.5	1 (ref)		1 (ref)		1 (ref)	
≥6.5	1.18 (0.573, 2.41)	0.659	2.312 (0.728, 7.35)	0.155	0.745 (0.291, 1.91)	0.538
25-(OH) Vitamin D (ng/ml)						
<20	1 (ref)		1 (ref)		1 (ref)	
≥20, <30	0.765 (0.508, 1.15)	0.200	0.751 (0.417, 1.35)	0.338	0.772 (0.425, 1.40)	0.396
≥30, <40	0.557 (0.315, 0.984)	**0.044**	0.754 (0.368, 1.55)	0.440	0.358 (0.138, 0.93)	**0.035**
≥40	0.720 (0.293, 1.77)	0.474	0.754 (0.214, 2.65)	0.659	0.867 (0.232, 3.23)	0.831

NKA, natural killer cell activity.

P values in bold denote statistical significance at the P < 0.05 level.

Compared to lack of physical exercise, low exercise (OR: 0.529; 95% CI: 0.299, 0.939; *P* = 0.030) and medium to high exercise (OR: 0.522; 95% CI: 0.277, 0.981; *P* = 0.043) decreased the risk of very low NKA in women (**Table 2**). In men, physical exercise was not associated with the risk (**Table 2**).

**Figure 5:**
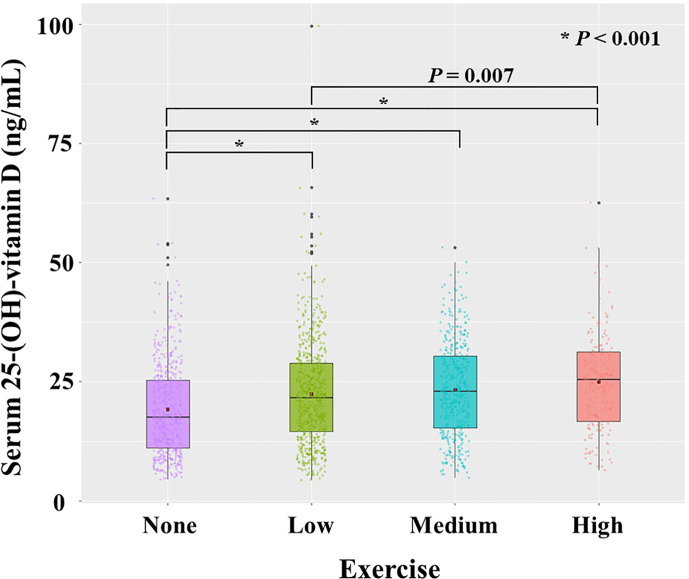
Distribution of serum level of 25-(OH)-vitamin D according to exercise. Each dot represents the individual value of 25-(OH)-vitamin D. Box plots show group medians, interquartile range (IQR), and spread of data with outliers for each group. A dot in each box plot shows the mean value. *P* values were calculated with student *t* test.

We checked the correlation between 25(OH)D (continuous variable) and exercise (categorical variable). 25(OH)D had a significant correlation with exercise (*P* < 0.001, r = 0.173). The mean value of 25(OH)D level increased gradually from the no-exercise group (19.2 ± 9.92 ng/mL) to low exercise (22.4 ± 10.8 ng/mL), medium exercise (23.3 ± 10.0 ng/mL), and high exercise group (24.9 ± 10.5 ng/mL) (**Figure 5**).

To investigate the independent association of vitamin D with NKA, we performed multiple logistic regression analyses in subgroups divided by exercise (**Table 3**). In the subjects who did not exercise, 25(OH)D 20–29.9 ng/mL decreased the risk of very low NKA (OR: 0.449; 95% CI: 0.202, 0.998; *P* = 0.049).

**Table 3:** Factors associated with very low NKA < 100 pg/mL compared to normal NKA ≥ 500 pg/mL in subjects who do or do not exercise.

	Subjects who do not exercise		Subjects who exercise	
	OR (95% CI)	*P* value	OR (95% CI)	*P* value
Gender				
Women	1 (ref)		1 (ref)	
Men	0.579 (0.306, 1.10)	0.094	1.19 (0.757, 1.86)	0.457
Age (years)				
18-40	1 (ref)		1 (ref)	
41-50	1.01 (0.476, 2.12)	0.990	1.05 (0.540, 2.03)	0.892
51-60	1.25 (0.553, 2.84)	0.589	1.18 (0.615, 2.24)	0.625
61-70	3.34 (1.19, 9.40)	**0.022**	1.23 (0.557, 2.70)	0.612
≥ 71	21.0 (1.63, 271)	**0.020**	2.38 (0.879, 6.47)	0.088
Physical exercise				
Low exercise	–		1 (ref)	
Medium to high exercise	–		0.801 (0.509, 1.26)	0.337
HbA1c (5)				
<6.5	1 (ref)		1 (ref)	
≥6.5	1.26 (0.328, 4.87)	0.733	1.18 (0.495, 2.79)	0.714
25-(OH) Vitamin D (ng/ml)				
<20	1 (ref)		1 (ref)	
≥20, <30	0.449 (0.202, 0.998)	**0.049**	0.953 (0.579, 1.57)	0.851
≥30, <40	0.783 (0.297, 2.06)	0.620	0.513 (0.252, 1.04)	0.066
≥40	1.59 (0.364, 6.90)	0.540	0.530 (0.155, 1.81)	0.311

NKA, natural killer cell activity; OR, odds ratio; CI, confidence interval; ref, reference.

P values in bold denote statistical significance at the P < 0.05 level.

### NKA and Age

In men, older-age was associated with increased risk of very low NKA (**Table 2**). Compared to age 18-40 years, the age-associated risk in men was stepwise increase from age 51–60 years (OR: 3.74; 95% CI: 1.53, 9.12; *P* = 0.004) to age 61–70 years (OR: 5.59; 95% CI: 2.00, 15.6; *P* = 0.001) and age ≥ 71 years (OR: 12.4; 95% CI: 3.62, 42.7; *P* < 0.001) (**Table 2**). On the other hand, women did not show the age-associated changes in risk (**Table 2**).

In men with age ≤ 60 years (age 18–60 years), 25(OH)D level 30–39.9 ng/mL significantly decreased the risk of very low NKA (OR: 0.112; 95% CI: 0.0147, 0.855; *P* = 0.035) (**Table 4**). In women with age ≤ 60 years, there was no significantly associated factor.

**Table 4:** Factors associated with very low NKA < 100 pg/mL compared to normal NKA ≥ 500 pg/mL in subjects with age ≤ 60.

	Total population (age ≤ 60)		Women (age ≤ 60)		Men (age ≤ 60)	
	OR (95% CI)	*P* value	OR (95% CI)	*P* value	OR (95% CI)	*P* value
Gender						
Women	1 (ref)		–		–	
Men	0.697 (0.460, 1.06)	0.089	–		–	
Age (years)						
18-40	1 (ref)		1 (ref)		1 (ref)	
41-50	1.08 (0.656, 1.76)	0.775	0.859 (0.473, 1.56)	0.619	1.86 (0.725, 4.80)	0.196
51-60	1.17 (0.694, 1.97)	0.558	0.547 (0.268, 1.12)	0.097	3.50 (1.40, 8.74)	**0.007**
Physical exercise						
Lack of exercise	1 (ref)		1 (ref)		1 (ref)	
Low exercise	0.862 (0.540, 1.38)	0.535	0.654 (0.357, 1.20)	0.169	1.31 (0.600, 2.88)	0.495
Medium to high exercise	0.676 (0.396, 1.15)	0.150	0.616 (0.309, 1.23)	0.169	0.920 (0.379, 2.23)	0.853
HbA1c (%)						
<6.5	1 (ref)		1 (ref)		1 (ref)	
≥6.5	0.984 (0.338, 2.87)	0.977	2.93 (0.748, 11.5)	0.123	0.289 (0.0375, 2.23)	0.233
25-(OH) Vitamin D (ng/ml)						
<20	1 (ref)		1 (ref)		1 (ref)	
≥20, <30	0.673 (0.417, 1.09)	0.105	0.747 (0.393, 1.42)	0.374	0.603 (0.288, 1.26)	0.180
≥30, <40	0.496 (0.246, 1.00)	**0.050**	0.803 (0.367, 1.76)	0.583	0.112 (0.0147, 0.855)	**0.035**
≥40	1.127 (0.419, 3.03)	0.813	1.23 (0.340, 4.41)	0.756	1.28 (0.258, 6.31)	0.765

NKA, natural killer cell activity; OR, odds ratio; CI, confidence interval; ref, reference.

P values in bold denote statistical significance at the P < 0.05 level.

In subjects with age > 60 years, men had a higher risk of very low NKA than women (OR: 2.48; 95% CI: 1.01, 6.08; *P* = 0.048) (**Table 5**). There was no significant difference between age 61–70 years and age > 70 years in the risk of very low NKA (**Table 5**). Compared to lack of exercise, low exercise (OR: 0.154; 95% CI: 0.050, 0.473; *P* = 0.001) and medium to high exercise (OR: 0.180; 95% CI: 0.060, 0.537; *P* = 0.002) decreased the risk significantly (**Table 5**). These associations were observed both in men and women with age > 60 years in separate subgroup analyses (**Table 5**).

**Table 5:** Factors associated with very low NKA < 100 pg/mL compared to normal NKA ≥ 500 pg/mL in subjects with age > 60.

	Total population (age >60)		Women (age >60)		Men (age >60)	
	OR (95% CI)	*P* value	OR (95% CI)	*P* value	OR (95% CI)	*P* value
Gender						
Women	1 (ref)		–		–	
Men	2.48 (1.01, 6.08)	0.048	–		–	
Age (years)						
61-70	1 (ref)		1 (ref)		1 (ref)	
≥ 71	2.04 (0.797, 5.24)	0.137	1.62 (0.267, 9.88)	0.599	2.43 (0.788, 7.50)	0.122
Physical exercise						
Lack of exercise	1 (ref)		1 (ref)		1 (ref)	
Low exercise	0.154 (0.0501, 0.473)	**0.001**	0.108 (0.0144, 0.810)	**0.030**	0.188 (0.0423, 0.836)	**0.028**
Medium to high exercise	0.180 (0.0603, 0.537)	**0.002**	0.180 (0.0323, 1.00)	**0.050**	0.191 (0.0441, 0.828)	**0.027**
HbA1c (%)						
<6.5	1 (ref)		1 (ref)		1 (ref)	
≥6.5	1.06 (0.340, 3.33)	0.915	2.45 (0.219, 27.4)	0.468	0.861 (0.240, 3.09)	0.819
25-(OH)-Vitamin D (ng/ml)						
<20	1 (ref)		1 (ref)		1 (ref)	
≥20, <30	1.02 (0.372, 2.82)	0.964	0.662 (0.127, 3.46)	0.625	1.48 (0.379, 5.80)	0.572
≥30, <40	0.834 (0.266, 2.62)	0.755	0.573 (0.0894, 3.67)	0.557	1.22 (0.264, 5.59)	0.802
≥40	0.312 (0.0342, 2.84)	0.301	–		0.922 (0.0750, 11.3)	0.949

NKA, natural killer cell activity; OR, odds ratio; CI, confidence interval; ref, reference.

P values in bold denote statistical significance at the P < 0.05 level.

## Discussion

We investigated how age, gender, vitamin D, and physical exercise were associated with immune function determined by NKA. The association of each factor with NKA varied widely according to age and gender. Age was the determining factor for the very low NKA in men but not in women. Sufficient level of vitamin D reduced the risk for very low NK in men, and physical exercise reduced the risk in women. In men with age < 60 years, sufficient level of vitamin D reduced the risk for the very low NKA. In the subjects with age ≥ 60, physical exercise reduced the risk both in men and women. DM is well-known to diminish immune function, but neither DM nor hyperglycemia represented by Hb A1c showed significant association with NKA when adjusted for other factors. To the best of our knowledge, this is the first observation demonstrating age- and gender-specific association of vitamin D and physical exercise with NKA.

Vitamin D plays an important role in immune health by affecting the maturation and differentiation of various immune cells, inducing production of antiviral peptide cathelicidins and defensins, and reducing production of pro-inflammatory cytokines (34, 35). Thus, vitamin D insufficiency [i.e., serum 25(OH)D level 20–29.9 ng/mL] or deficiency [serum 25(OH)D level < 20 ng/mL] are associated with increased infection susceptibility, increased cancer incidence, poor survival of cancer patients, and development of autoimmune diseases (34, 36, 37). For example, recent studies suggested that vitamin D insufficiency and deficiency were associated with increased susceptibility to COVID-19 and severe presentation and mortality from it (38–40). Vitamin D, having an immune modulatory function, is considered to reduce the production of pro-inflammatory cytokines, thus, reducing the severity and mortality of COVID-19 (35). Therefore, in case of vitamin D deficiency, vitamin D supplementation or rapid correction of 25(OH)D level with high-dose regimens were recommended by experts (35, 41, 42). For normal calcium homeostasis and bone health, many studies showed that serum 25(OH)D around 20 ng/mL is required (43). Yet, the optimal concentration of vitamin D for proper immune reactions has not been determined. For normal immune function, it is considered that much higher level than 25(OH)D 20 ng/mL is required (36). *In vitro*, 65 ng/mL 25(OH)D has been found to be more effective than 30 ng/mL or 90 ng/mL in inducing human NK cell-mediated antibody-dependent cell cytotoxicity (44). In adults, 25(OH)D ≥ 38 ng/mL, compared to lower levels, was associated with lower incidence of acute viral respiratory infection (45). In women, 25(OH)D ≥ 40 ng/mL was associated with lower risk of invasive cancer (46). On the other hand, serum 25(OH)D level revealed a U-shaped association with all-cause mortality. The all-cause mortality was the lowest with serum 25(OH)D levels of 30–40 ng/mL in a general population comprising of 26,916 European individuals (47) and a hospitalized population comprising of 24,094 adult patients (48). Our results indicated that the serum 25(OH)D level of 30–40 ng/ml might be optimal for the NKA. The correction target 40–60 ng/mL against COVID-19 suggested by some experts (41) may have directed in terms of immune modulatory function. Our results demonstrated that vitamin D supplementation might be a good strategy to take care of immune health. Albeit, caution should be taken in vitamin D supplementation because excessive vitamin D may cause disturbance of calcium metabolism.

Physical exercise is a well-known factor that enhances immune function. Many studies reported that physical exercise improved health outcome of cancer patients and ameliorated immunosenescence in the elderly population (49–53). A meta-analysis demonstrated that higher level of habitual physical activity is associated with reduced risk of community-acquired infectious disease and mortality by it (54). Also, physical activity interventions resulted in improved state of immune markers: increased CD4 cell counts, increased salivary immunoglobulin IgA concentration, decreased neutrophil counts, and higher antibody concentration after vaccination (54). One more important factor between the physical exercise and immune function is the intensity or duration of the exercise. In an animal study, mice infected with influenza showed significantly higher survival with moderate exercise, but prolonged exercise had a worse outcome than that of the control, although it was not statistically significant (55). Similar findings were observed in humans. Early observations suggested that athletes who were subjected to strenuous exercise were more prone to getting infectious diseases during outbreaks of the infectious diseases (56). Following studies demonstrated that immune function increased immediately after maximal exercise but depressed afterwards, and it took some time for the immune function to fully recover (57–60). Based on these findings, it was hypothesized that there exists an “open window” for opportunistic infection lasting up to 72 hours after a prolonged endurance exercise, so repeated strenuous exercise without adequate recovery may prolong the “open window” and lead to impaired immune function (57, 61). Therefore, moderate physical exercise in intensity and duration followed by adequate recovery time should be emphasized in lifestyle management. The current study demonstrated that the lack of physical exercise was associated with the risk of very low NKA. This was consistent with a previous study which showed that physical inactivity was associated with decreased NKA (62). Furthermore, our study suggested that physical exercise may be immunologically more beneficial to women.

As life expectancy increases, strategies to enhance healthy life expectancy are of attention (63, 64). As human body ages, immune function also ages, so called immunosenescence. Immunosenescence results in persistent low-grade inflammation, autoimmune diseases, allergic diseases, poor vaccine responses, increased susceptibility to severe infections, increased cancer incidence, and high mortality in the elderly (65–70). Research effort began as early as 1980’s to find immune characters that define healthy longevity. A Swedish longitudinal cohort study with octogenarians and nonagenarians revealed that a combination of immune characters (inverted CD4^+^:CD8^+^ ratio (< 1), poor T-cell proliferation response to mitogens *in vitro*, and increased number of CD8^+^ T lymphocytes representing effector-memory T cells) was associated with mortality (6–8). However, this trend was not reproduced in more recent studies conducted in other countries (10, 11). In our study, we presented age-associated changes in NKA: older-age increased the risk of the very low NKA in men. Compared to adaptive or other innate immune cells, NK cell function is better preserved along the immunosenescence process owing to the reciprocal increase in NK cell counts to compensate per-cell functional decrease (71, 72). Thus, NKA or NK cell counts may serve as a good parameter of immunosenescence or healthy immune aging. Further, the present study showed that physical exercise reduced the risk for very low NKA in individuals with age ≥ 60 years. This is consistent with previous studies that investigated the role of physical exercise in ameliorating immunosenescence. For example, moderate cardiovascular exercise in healthy older adults resulted in increased seroprotection after influenza vaccination while balance and flexibility intervention did not (73), and low-dose combined resistance and endurance training for 6 weeks in the elderly resulted in improvements in immune markers: an increase of the CD4+/CD8+ T cell ratio and decrease in systemic levels of interleukin (IL-) 6, IL-8, IL-10 and vascular endothelial growth factor (VEGF) (74). Recent knowledge indicates that there may be a cause and an effect relationship between physical exercise and health outcomes mediated by enhanced immune function (53). It is discussed that the key role in that relationship may be played by skeletal muscle that the muscle takes an immune regulatory function by producing myokines, that is cytokines released by muscle cells (53). Thus, avoiding sarcopenia by physical exercise may serve as a potential strategy to delay immunosenescence in the elderly (53).

Generally, women live longer and are healthier than men (63). This gender-dependent life expectancy can be closely linked to a gender-specific difference in immunity that is attributed to lifestyle and biological factors (75). Women carry two X chromosomes containing many genes related to immunocompetence, including Toll-like receptors, cytokine receptors, immune-response-related proteins, and transcriptional and translational control effectors (76). In contrast, the Y chromosome contains male-specific inflammatory genes (77). Also, sex hormones affect immune cells differently. Estradiol is immune-enhancing, whereas testosterone is immune-suppressive (75). With advancing age, decline in adaptive immune parameters is greater in men (75), and reciprocal increase in NK cells is greater in women (78). In our study, NKA decreased with age in men but not in women. This supports that NK cells compensate immunosenescence better in women than in men.

This study has some limitations. It was a retrospective study conducted at a single health check-up center that it may have selection bias. The questionnaire used in the health check-up did not ask the intensity of the physical exercise but the frequency and duration only. The population size was not large enough to allow significant results in all meaningful factors including hyperglycemia. Nevertheless, we analyzed the data as accurately as possible and obtained several novel insights on gender, age, exercise, vitamin D, and immune function.

The present study demonstrated that vitamin D reduced the risk of very low NKA in men, and physical exercise did so in women and in individuals with age ≥ 60 years. Age was a major risk factor of very low NKA in men but not in women. Thus, there were gender- and age-dependent differences in factors that was associated with NK cell immune function. We hope these findings help to delineate an individulized strategy to enhance immune function in the immunologically normal population.

## Data Availability Statement

The datasets presented in this article are not readily available because the data pertains to our institute, and further analyses are now being undertaken. Requests to access the datasets should be directed to SO, ohsooyoun@hotmail.com.

## Ethics Statement

The studies involving human participants were reviewed and approved by CHA Bundang Medical Center. Written informed consent for participation was not required for this study in accordance with the national legislation and the institutional requirements.

## Author Contributions 

SO, SC, and S-WC contributed to the study design, data collection, study analysis, manuscript writing, critical review of the manuscript, and final approval of the manuscript submission. SH, JK, YC, JL, and KK assisted in analyzing and interpreting the data, critical review of the manuscript. All authors contributed to the article and approved the submitted version.

## Funding

This work was supported by the National Research Foundation of Korea (NRF) grant funded by the Korea government (MSIT) (No. 2020R1F1A107714812).

## Conflict of Interest

The authors declare that the research was conducted in the absence of any commercial or financial relationships that could be construed as a potential conflict of interest.
